# Establishing and testing a robot-based platform to enable the automated production of nanoparticles in a flexible and modular way

**DOI:** 10.1038/s41598-023-38535-6

**Published:** 2023-07-15

**Authors:** Sofia Dembski, Thomas Schwarz, Maximilian Oppmann, Shahbaz Tareq Bandesha, Jörn Schmid, Sarah Wenderoth, Karl Mandel, Jan Hansmann

**Affiliations:** 1 Fraunhofer Institute for Silicate Research ISC, Neunerplatz 2, 97082 Würzburg, Germany; 2 Department of Tissue Engineering and Regenerative Medicine TERM, University Hospital Würzburg, Röntgenring 11, 97070 Würzburg, Germany; 3 Goldfuß Engineering GmbH, Laboratory Automation, 72336 Balingen, Germany; 4 Department of Chemistry and Pharmacy, Friedrich-Alexander University Erlangen-Nürnberg (FAU), 91058 Erlangen, Germany; 5 Faculty of Electrical Engineering, University of Applied Sciences Würzburg-Schweinfurt, 97421 Schweinfurt, Germany

**Keywords:** Automation, Design, synthesis and processing

## Abstract

Robotic systems facilitate relatively simple human–robot interaction for non-robot experts, providing the flexibility to implement different processes. In this context, shorter process times, as well as an increased product and process quality could be achieved. Robots short time-consuming processes, take over ergonomically unfavorable tasks and work efficiently all the time. In addition, flexible production is possible while maintaining or even increasing safety. This study describes the successful development of a dual-arm robot-based modular infrastructure and the establishment of an automated process for the reproducible production of nanoparticles. As proof of concept, a manual synthesis protocol for silica nanoparticle preparation with a diameter of about 200 nm as building blocks for photonic crystals was translated into a fully automated process. All devices and components of the automated system were optimized and adapted according to the synthesis requirements. To demonstrate the benefit of the automated nanoparticle production, manual (synthesis done by lab technicians) and automated syntheses were benchmarked. To this end, different processing parameters (time of synthesis procedure, accuracy of dosage etc.) and the properties of the produced nanoparticles were compared. We demonstrate that the use of the robot not only increased the synthesis accuracy and reproducibility but reduced the personnel time and costs up to 75%.

## Introduction

Process automation is a trend in the twenty-first century. Key boosters for this are increasing cost pressure, high personnel costs and laboratory equipment (keyword: "release of personnel capacities"), the acceleration of workflows and the resulting faster processing of analyses, better quality management, high regulatory requirements in the manufacturing as well as a broader availability of production technologies for the end user. In this way, automation can additionally help to counteract the lack of skilled labor, that growth in recent years especially in Europe^1^. There are different levels of automation^2^. At the lowest level of automation, the laboratory staff performs all working steps. The advantages are a high degree of flexibility and the cognitive abilities of the human to enable complex and varied activities. The highest level of automation is a fully automated process. The development of such a process entails high investment costs and can thus be economically feasible only in the case of an extremely high throughput. This requires a completely stable, safeguarded, low-variant production process with a high outlay of operation, maintenance and troubleshooting. Various levels of partial automation are in between manual execution and full automation, in which only specific sub-processes are automated.

When putting a focus on nanoparticles (NPs), it can be constituted that in the recent years, the control and reproducibility of NP syntheses have improved by using partial automation such as differently designed reactors (e.g., batch reactors or liquid handling devices)^2–13^. In particular, microfluidic-based solutions have become established, e.g., for the fabrication of gold-, semiconductor-, lipid-based NPs as well as NP-based RNA/DNA drug delivery systems^14–18^. Partially automated workstations, known from pharmaceutical production, are also applied in NP syntheses. However, the main application of such systems is a high-throughput screening, where several parameters or substances can be varied and tested in a short time^19^. Hereby, optimal synthesis conditions can be determined. In addition, several NP variants with the desired properties can be provided according to the requirement profiles^19^. It is noteworthy that these systems neither enable the fully-automated production of NPs nor do they support a broad application domain.

NPs are suitable candidates for various commercial and domestic applications, which include catalysis, imaging, medical applications, energy-based research, and environmental applications^20^. One of the biggest challenges in the NP synthesis, is to establish a scalable manufacturing process that ensures reproducible product qualities in order to meet the standard requirements. However, NP functionality depends on the size, composition, shape and structure. An automated process could increase the scalability, quality and versatility of developed NP systems and thereby foster the development of NP applications. Moreover, a continuous digital documentation of the process parameters and measurement results during the synthesis meets regulatory requirements for documentation. Despite these advantages, the transfer of manual syntheses into fully automated production has not been achieved yet^21,22^.

In this work, we present the successful development of a robot-based infrastructure and an automation process for the reproducible production of silica NPs. The system supports an easy integration in a laboratory environment and benefits from the use of standard laboratory equipment. To enable a high flexibility, scalability, and simple adaption to different NP production processes, the system is designed in a modular concept. All devices and components of the automated system are optimized and assembled in such a way that all required movement sequences, speeds, positioning, signal and measurement parameters ensure a sufficiently accurate and reproducible production process.

For a proof-of-concept model system, silica NPs were chosen that were designated to act as building blocks for photonic crystals^23,24^. Photonic crystals represent a class of functional materials where a well-designed nano-property replaces less sustainable approaches to achieve the same functionality^25–28^. Concretely, a structural color is created due to nano building blocks, which could replace organic dyes that are vulnerable to degradation or inorganic toxic or rare elements that would otherwise yield the desired color^29^. Via a photonic crystal approach, in principle, simply the well-ordered arrangement of equally sized building blocks “does the trick” and yields the color. However, the requirement is that the size distribution of the building blocks, i.e., the NPs that make up the photonic crystal, is very small. This is exactly why photonic crystals are well suited for this study. Only if the explained goal of exact control of parameters and thus product outcome, i.e., herein an exact NP size is achieved, the final functionality is achieved. In other words: If no exact NP size can be produced, no structural color is obtained.

An already established protocol for the manual synthesis of silica NPs was available, which gave a particle size of 200 nm in diameter. The automated NP synthesis reproducibility was benchmarked against manual syntheses done by lab technicians. To this end, different processing parameters (time of synthesis procedure, the accuracy of the dosage etc.) and properties of produced NPs, e.g., size or polydispersity were compared.

## Results

### Automation process

**Figure 1:**
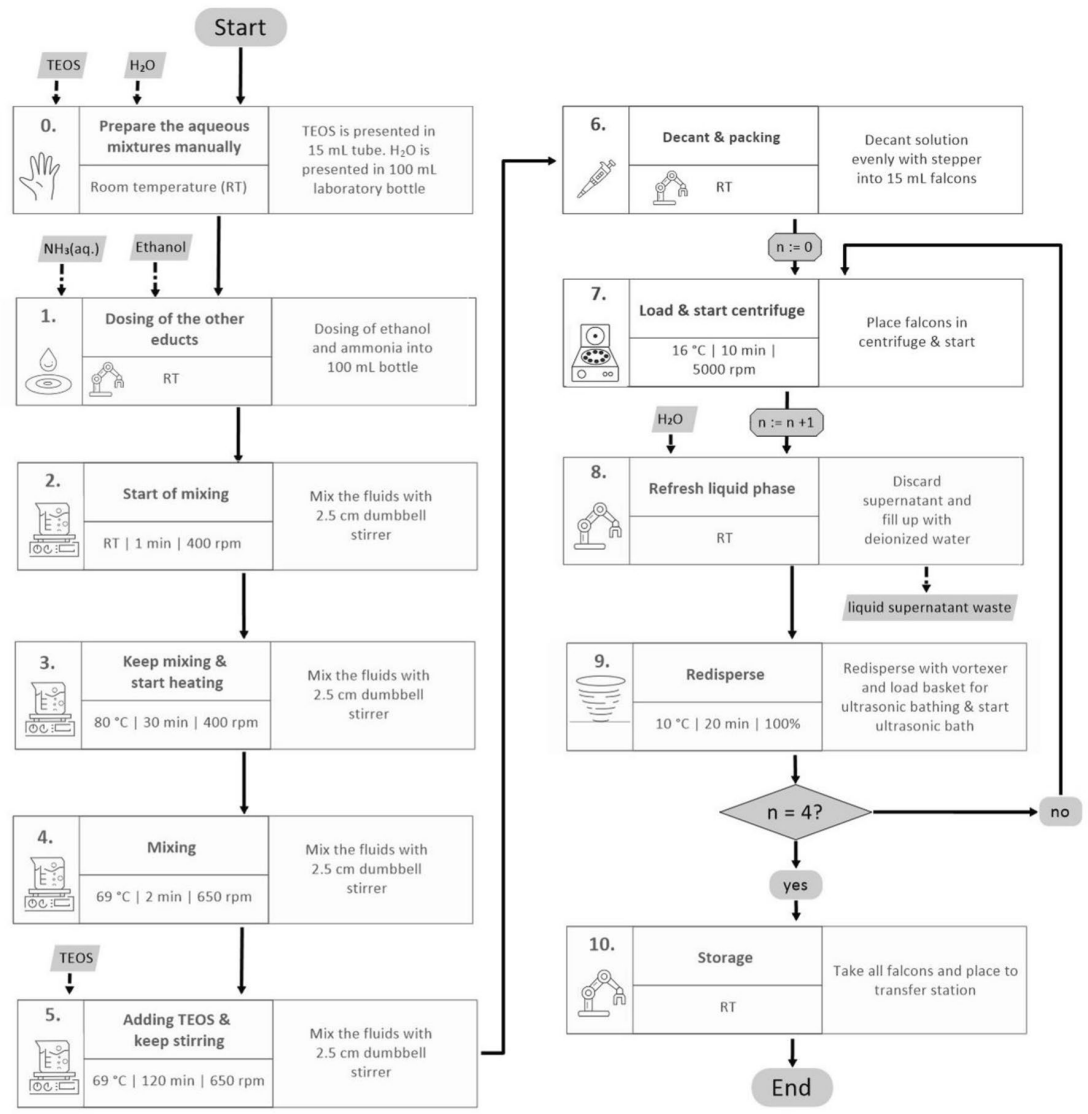
Diagram of the workflow for the automated synthesis of the silica NPs. Prior to the start of the synthesis, consumables and materials such as tetraethyl orthosilicate (TEOS), water, or ethanol are introduced into the robot platform. The derived workflow includes all process steps required for a complete synthesis of silica NPs and renders a translation of the standard operation procedures (SOP) into specific steps that process an input (information and material) into an output, which is the input for a consecutive process step. In addition to the process steps and required parameters, main components are shown.

At first, the manual silica NP synthesis process was analysed using the available standard operating procedure (SOP) as well as photo and video documentation. An exemplary documentation of the synthesis steps is shown in the Supplementary Materials (Figs. S1–S8). Figure 1 illustrates the resulted workflow for the automated synthesis procedure. According to the manual process, the automated synthesis includes the following general steps: dosing of the educts, mixing, heating, cleaning of prepared particles and storage. After the dosing of ethanol, water and aqueous ammonia, mixing is performed by magnetic stirring and followed by heating. Subsequently, tetraethyl orthosilicate (TEOS) is added to the solvents and the mixture is stirred for 2 h at 69 °C. Every synthesis step was assessed and the required process parameters were derived. Possible sources of error and tolerances in weighing of solids, dosage of liquids, the temperature ranges and the duration of the individual steps were identified and specified. In a sensitivity analysis, the individual impact of each possible deviation was derived in order to identify tolerances that ensure a proper NP synthesis. Some synthesis steps needed to be modified for the translation into an automated process, e.g., selection of synthesis vessels, preparation of starting substances, dosing of solvents, duration of synthesis. To accelerate the heating of the heating block inside of the robot infrastructure, the temperature of the heating stirrer was adjusted to 80 °C for 30 min during the preheating (Fig. 1, step 3). In the manual synthesis, the NP growth is performed at 60 °C. To obtain the needed temperature inside of the glass bottle during the automated production, the temperature of the heating stirrer was adjusted to 69 °C during the NP growth step (Fig. 1, step 4 and 5). Similar to the manual procedure, the NPs were cleaned by centrifugation and washed four times with deionized water (Fig. 1 steps 6–9).

**Figure 2:**
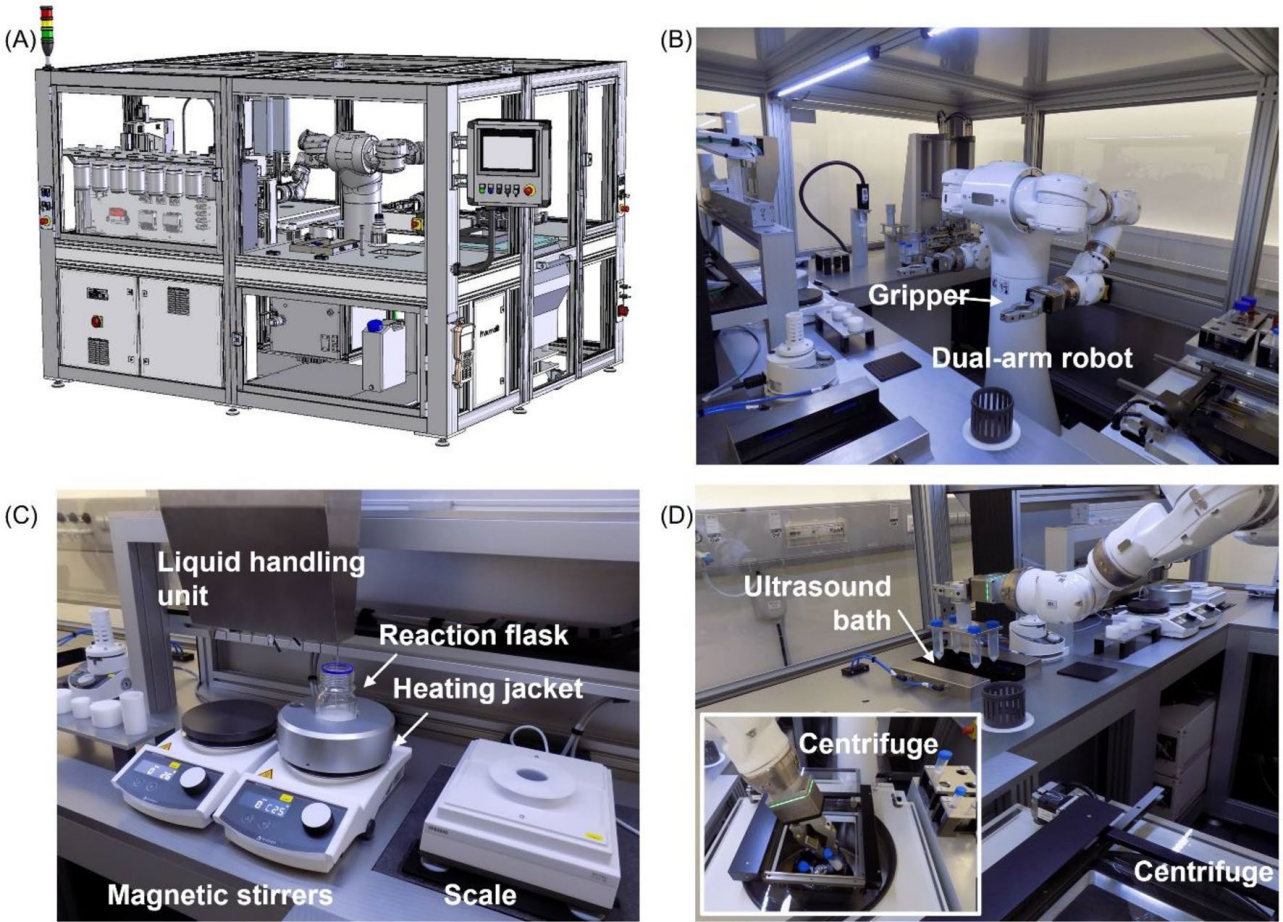
Robot-based plant for the automated production of NPs and main components. (**A**) Computer aided design (CAD) model showing the installation of the dual-arm robot in a tailored housing made from aluminum profiles. The housing is closed and can be accessed through specific ports that facilitate maintenance and supply with materials. A human machine interface allows the user to control the system. (**B**) The dual-arm robot can interact with (**C**) a liquid handling station and (**D**) an automated centrifuge.

After the description of the general workflow and its evaluation, the process was further broken down into smallest functional steps, e.g., opening of a container or pipetting a liquid, and a robot cell was designed. This robot cell (Fig. 2A and Fig. S9) is housed using standard aluminum profiles and transparent polycarbonate plate materials. Inside the cell, a dual-arm robot (Fig. 2B), liquid handling unit (Fig. 2C), decapper, vortex and ultrasound device, and a centrifuge (Fig. 2D) were installed.

The robot is equipped with two linear electric grippers. The grippers feature force control, which allows to control the contact pressure and to ensure secure gripping. Thereby, the robot can handle all tools and materials during the synthesis and mainly serves as connecting link between the units inside the robot cell. Positions of the robot and movements were programmed in jobs that can be called by a programmable logic controller (PLC). The liquid handling unit enables the dosing of volumes from 1 mL to the volume corresponding to the total storage tank. For small volumes between 1 μL to 50 mL, an automated multistep pipette is available. In this case a 25 mL PD-Tip (Precision Dispenser Tip) is inserted. Alike the dosing unit, the pipette receives commands through a RS232 interface from the PLC. To purify solutions as required for process step 9 (Fig. 1, step 9), a specific centrifuge for automated processes (Fig. 2D, inset) and an ultrasonic bath (Fig. 2D) are installed, where centrifuge tubes can be introduced by the robot. The robot uses a tailored rack in which up to six centrifuge tubes can be placed.

**Figure 3:**
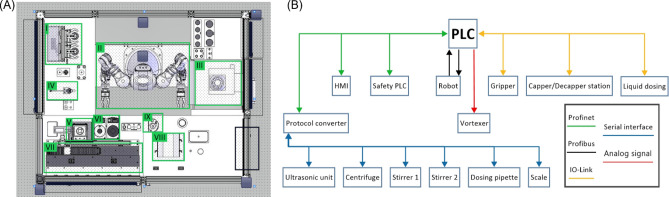
System architecture. (**A**) The human machine interface is implemented as touch screen and allows to access the main components of the system in a graphical user interface. The interface facilitates setting the parameters of a synthesis, starting the system and visualizing parameters on the system and on a device level: (I) Station for vessels with rotary thread, (II) 2-arm robotic manipulator MOTOMAN CSDA10F, (III) centrifuge, (IV) multistep pipette, (V) precision balance, (VI) heating stirrer, (VII) dosing station for liquid media, (VIII) ultrasonic device, (IX) vortexer. (**B**) These components communicate through different buses and use different communication protocols. The central component that instructs the peripheral devices is a PLC.

An external human machine interface (HMI) enables to access the platform and monitor synthesis progress or to enter process parameters. The main page of the graphical user interface shows all units of the robot cell (Fig. 3A). Using the touch screen function, a user can access the unit and monitor the state of the device. Moreover, process parameters are shown and can be entered. These parameters are forwarded to the PLC that is the main control unit in the system (Fig. 3B). The PLC features multiple communication protocols and thereby allows to connect all components in the unit. Inside the PLC, the workflow for the synthesis of silica NPs (Fig. 1) is implemented in terms of a step sequence. Based on this step sequence, the PLC starts and stops the functional devices and calls the robot jobs to transfer materials inside the cell.

### Particle synthesis: robot vs human (lab technicians)

**Figure 4:**
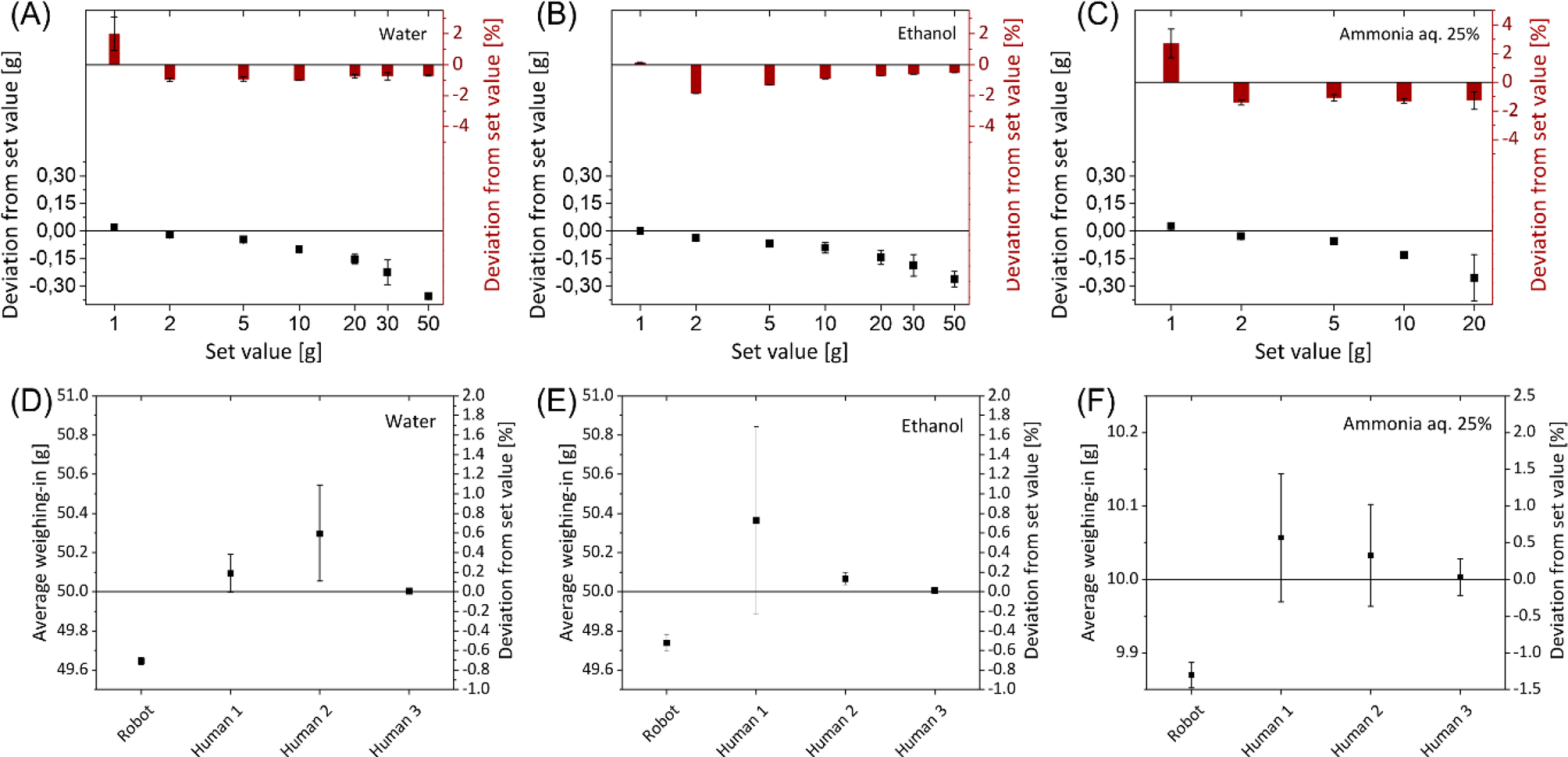
Comparison of the accuracy in liquid handling of different solvents. (**A**–**C**) Automated and (**D**–**F**) manual dosing. Human 1–3 represent three different lab technicians with varying levels of lab experience.

The NP synthesis is very sensitive to deviations between a required volume of a reactant and the actual volume used. Thus, the accuracy of the dosing unit was assessed. Considering the relevant volume ranges of 1–50 g for water and ethanol as well as 1–20 g for the aqueous ammonia solution, liquids were dispensed at least as accurate as in the manual process. To improve the initial accuracy, a characteristic curve was setup and the deviation between setpoint and actual volume was decreased by taking the inverse of the characteristic curve. Thereby, the real dispensed volume was linked to an adapted setpoint. As a result, relatively small deviations for water, ethanol and aqueous ammonia solution were achieved (Fig. 4A–C). Moreover, the automated dosing was compared to the dosing accuracy in the manual process. According to the synthesis conditions, three different people (laboratory technicians) dosed 50 mL of water, 50 mL of ethanol and 10 g of an aqueous ammonia solution (Fig. 4D–F). The dosing experiment was performed in a triplicate run for every liquid. The deviation for a chosen setpoint was less in the automated compared to the manual synthesis. The comparison of the manual performance and the performance using the robot system shows significant differences. The deviation between single manual syntheses strongly depends on the human who performs the process.

Both, the robot and lab technicians have more difficulties to dose ammonium, due to higher volatility of this liquid. To overcome the problem with volatile components, further process solution should be developed e.g., a closed reaction vessel system.

**Figure 5:**
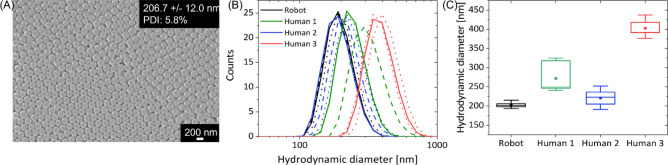
Characterization of manually and automated produced silica NPs. (**A**) SEM image of robot-synthesized NPs. (**B**) Comparison of size distributions obtained by dynamic light scattering measurements on dispersed nanoparticles either prepared manually or via the robot. (**C**) Average hydrodynamic NP diameter and deviation of single experiments.

To demonstrate the advantage of automation, NP manufacturing was performed by a robot and three different lab technicians with varying levels of lab experience, and in each individual case the syntheses were repeated three times. Figure 5A exemplarily shows the SEM image of the automated synthesized NPs. Obtained NPs appeared in spherical shape. The mean NP diameter was determined to be 206.7 ± 12.0 nm with a polydispersity index (PDI) of 5.8%. This data was obtained by manually measuring the particle sizes of 100 particles in the scanning electron microscopy (SEM) images.

The hydrodynamic diameter measured by dynamic light scattering (DLS) showed for all synthesis, manual and automated, a Gaussian-like distribution. The manual synthesis, which was performed by three different lab technicians (every person three repetitions), revealed that there can be deviations between different batches for an experimenter, e.g., experimenter 1 (Fig. 5B). Due to precisely controlled automated process steps such as the dosing of liquids or the adherence to the present timing and temperatures, small deviations between synthesized particles and between different batches of NPs were measured. Additionally, the mean values of the measured diameter distribution varied between the individual lab technicians, whereas the mean values for the automated synthesis were consistent. The comparison of the measured mean values of each experiment confirmed the finding from the NP size distribution (Fig. 5C). All three batches of NPs from automated production have a monodisperse size distribution that results in a consistent blue color after drying the NP suspension (Fig. S10). However, if the primary NPs are polydisperse, no self-assembly is possible and thus no coloration is apparent. This effect is only possible due to the reproducibility of NP characteristics.

**Figure 6:**
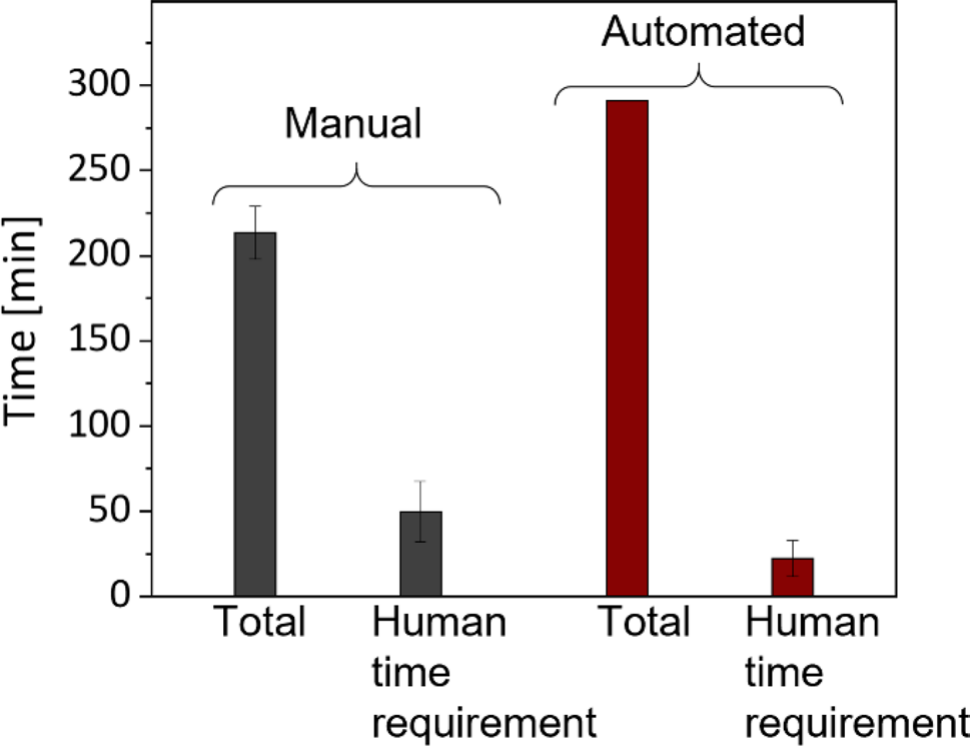
Time required for one silica-NP synthesis, automated syntheses and the total synthesis time (from the start of the process to obtaining the final product).

To assess the economic aspect of the synthesis automation, the time and costs needed for manual and automated synthesis were determined. Figure 6 shows the comparison of the time spent by lab technicians for manual and automated syntheses as well as the total synthesis time (from the beginning of the process to the obtaining of the final product).

In the case of the manual process, the total time includes time spent by lab technicians for the lab work and time needed for the sample preparation for lab devices without human participation (centrifugation, stirring, ultrasonic redispersion). Absolute personnel time during the manual process is in the range between 34 and 66 min, depending on the staff member’s skills and lab experience. Automated total synthesis time includes time of a staff member needed for the preparation of the robot-plant (filling the storage containers, setting of educts etc.) and the working time of the robot and all infrastructure devices during the work flow. The total time of the automated synthesis is approx. 80 min longer than manual synthesis. The reason for this is that the robot and the devices connected to it need some lead time. In contrast, the time spent by humans is approx. 44% shorter. The average time spent by a staff member during the automated synthesis is about 22 min. The longest time that a staff member need is a maximum of 30 min. It is required for the preparation of stock solutions and refilling of the plant, which usually takes place only 1–2 times a month, according to our initial estimates. Once the plant is filled, synthesis can be started immediately after calibration. This usually takes max. 15 min. The personnel costs are usually related to the time spent. With the reduction of the human time required, the personnel costs can be reduced by 75% per synthesis, assuming that the synthesis costs arise from the time spent of personnel for one synthesis process. To note, the automated systems facilitate 24/7 and using a sophisticated planning module, the throughput can be increased. Additionally, the system covers many process steps that are also required in different NP synthesis. This makes an adaption to other NP applications possible.

## Discussion

A dual-arm robot-based infrastructure for the reproducible production of NPs was developed. The robot-based plant has a modular concept and includes standard laboratory equipment. We demonstrated that dual-arm robots can offer a flexible solution combined with all the advantages of automation, such as extreme repeatability and programmability of different complex process steps. To demonstrate the feasibility, an automated process for the synthesis of monodisperse silica NPs was established. All devices and components of the automated system were optimized and adapted according to the synthesis conditions. The resulted robot-based plant is suitable for all wet chemical syntheses involving steps such as mixing, centrifugation and ultrasonication. Depending on the synthesis, it is possible to expand this plant infrastructure with various additional modules (heated reactor, microfluidics, particle size analyzer etc.) to increase the process flexibility.

To demonstrate the benefit of the automated NP production, manual and automated syntheses were benchmarked. To this end, different processing parameters (time of synthesis procedure, the accuracy of the dosage etc.) and properties of the produced NPs, e.g., size or polydispersity were compared. We demonstrated that the use of the robot not only increases the synthesis accuracy and reproducibility but reduces the personnel costs. The advantages of automated synthesis are particularly evident when compared with syntheses performed manually by different people. The comparison shows that the reproducibility of the NPs (NP size and size distribution) obtained after the synthesis is highly dependent on the qualification of the operator. Deviations in the obtained product are thus faced, as in a daily situation in the laboratory, syntheses may be carried out by different people depending on who is available at the time.

In the present study, the human technicians used the same instruments as the robot. Thus, the resulting difference in NP size and size distribution is mainly due to variations in the timing and speed of operational steps, such as the timing and speed of TEOS addition. Since the robot always performs this addition in the same way, the resulting NPs are quite similar. In contrast, human operators are sometimes a little faster or slower, or more of the liquid ends up on the reaction vessel wall. All these small changes can affect the resulting size of the NPs as well as their size distribution, since the nucleation process is one of the most critical and decisive steps in this model synthesis—a problem that clearly applies to most NP syntheses in general.

Due to their design, dual-arm robots largely correspond to human kinematics. Such robots implement protocols and work steps according to manual processes, can handle any common laboratory equipment and can thus be integrated into different production processes (fixed installation or mobile) with a minimum of effort^30–32^. The surrounding infrastructure can be flexibly designed according to the process requirements and the available space^33^. This means that automation can be carried out in a space-saving manner and without the necessity to build new laboratories and to invest into expensive plant technology. In addition, it accelerates of workflows and the resulting processing of analyses and enables better quality management regarding to high regulatory requirements in production ("Good manufacturing practice” (GMP) production). The safety cage that surrounds the automated lab increases worker safety from chemical exposure and provides the ability to create clean or sterile room conditions. The main challenge here will be the providing of a flexible and user-friendly control during robot integration. Ideally, laboratory staff should be able to operate the robotic system without any programming skills.

## Materials and methods

### Manual silica NP synthesis

The chemicals were used as purchased without further purification. The syntheses of silica NPs with a primary particle size of about 200 nm were carried out according to previously published protocols^34^: 3 g of deionized water were mixed with 7.2 g of aqueous ammonia solution (25 wt%, Merck, Germany) and 47.36 g of ethanol (99%, Jäckle-Chemie, Germany). This mixture was heated to 60 °C under vigorous stirring, before 5.6 g tetraethyl orthosilicate (TEOS, 99%, Sigma-Aldrich, Germany) were added. After 2 h of reaction time, the NP dispersion was allowed to cool to room temperature. Afterwards, NPs were collected by centrifugation and washed at least three times with deionized water (5000 rpm, 8 min). The syntheses were performed by three different lab technicians and were repeated three times each.

### Transfer of the manual synthesis to the automated process

The manual synthesis process for the production of silica NPs (d = 200 nm) was documented in detail. To this end, manual processes were recorded on videos and photos (see Supplementary Materials, Figs. S1–S8). Afterwards, the process was evaluated with regard to its automation and broken down into individual steps that could be implemented. In addition, necessary material supply was determined and the type and shape of vessels, suitable for automation, were specified. The substances required for the synthesis were assessed in terms of dosing accuracy. On this basis, concepts for identified individual process steps were designed.

The chemicals and mass quantities used for automated synthesis were identical with those used in the manual process. For automation, the manual NP synthesis was broken down into three main steps (Table 1).

**Table 1:** Steps of the automated particle synthesis.

Step number	Function	Rotation speed during mixing (rpm)	Time (min)	Temperature (°C)
1	Heat up	400	30	80
2	TEOS adding	650	2	69
3	Particle growth	650	120	69

The temperature and duration of the heating process were chosen so that a temperature of 60 °C is reached in the reaction vessel within the specified time. During the TEOS addition and the particle growth step, the temperature in the reaction vessel should be constant at 60 °C; this is given for a process temperature of 69 °C. Analogous to the manual synthesis process, the NPs were centrifuged three times and washed with deionized water (5000 rpm, 8 min). Each centrifugation step was followed by 2 min ultrasonic treatment each followed by a vortex step.

### Mechanical design of the plant

According to the analysis of the manual process, a CAD model of the plant was developed (Solidworks), where all devices and actuators are organized in the workspace of the robot. Therefore, the selection of devices was performed with respect to usability in the process, connectivity through a suitable communication interface, and automation capability. Grippers (Weiss Robotics CRG-Series) were identified and the fingers of the grippers were created to handle all materials during the process. Afterwards, the model of the robot plant was imported in a simulation software (MotoSim EG-VRC, Yaskawa) to assess the accessibility to all components in the cell. According to the envisioned throughput, the capacities and size of magazines and storage for solids and fluids were calculated. With respect to the actual location, local constraints were incorporated in the design of the plant, e.g., entry ways into building, doors, passageways, stairs, elevators; accessibility from all sides, power/media connections in room, doors, windows, hatches for maintenance and operation; placement of lighting, status/warning lights. Moreover, the position of control cabinets and pneumatic units and the accessibility of electrical main/emergency stop switches were considered in the installation plan.

### Assembly

To assemble the plant, lightweight, flexible, and powder coated aluminum profile constructions ST37 (ITEM) were used. The profiles have smooth surfaces with the possibility of sealing and closing joints when installed in a laboratory area. The profiles allowed the integrated routing of cables. Particular emphasis was laid on the industrial suitability. To close the robot cell, polycarbonate (Makrolon) plates were used for windows and doors. For interior fittings, aluminum components with required coatings (anodizing, hard coating), V2A/V4A stainless steel surfaces, drains, drying units and POM plastic in white or black for holders, trays, magazines were applied.

### Software and programming

First, the required workflow was derived and documented in step sequence (DIN EN 60848). A process analysis allowed to determine whether a programmable logic controller or an industrial PC (IPC with WinCC + software modules) should be used. Considered criteria were user management, audit trail, LIMS connection, database, metadata collection, work plans with scheduler. According to the analysis, programming of a programmable logic controller (PLC, Yaskawa Vipa System 300S+) was performed using Totally Integrated Automation Portal (TIA-Portal, Siemens). The identified relevant process steps in the step sequences were considered as function units. For the sub-programs, function units were programmed in a combination of function block diagram, flowchart and structured text. Peripheral devices such as liquid handling devices or the centrifuge were integrated into the system through communication interfaces such as RS232 or Profibus. Manufacturer information of protocols allowed the control of the peripheral devices by the PLC. For the visualization of the plant and the process, a human machine interface (TP1200 Comfort, Siemens) was installed. Windows control center (WinCC) enables the remote configuration and user interaction. In the HMI, menus, commands, parameters of the devices are visualized according to the need of a specific operator, i.e., admin, user, and service.

### Commissioning

Following the installation of the plant and the integration of all peripheral systems such as the liquid handling unit, the centrifuge and the ultrasonic water bath, the communication and functionality of each device was tested individually. Therefore, specific device parameters were set such as the rotational speed of the centrifuge. After the proof of functionality of each device in the plant, the total process workflow was conducted under detailed supervision, and modifications were done to achieve the fully automated production of the NPs. Before the documentation of the process and plant and the preparation of a manual and user instructions, safety tests and a Failure Mode and Effect Analysis (DIN EN 60812) was performed. To improve the throughput, process parameters such as incubation times were optimized as the 24/7 working mode of the plant offers a high degree of freedom in process scheduling.

### Human robot pipetting accuracy test

Three employees (human laboratory technicians with varying levels of lab experience) in comparison to the robot performed the human–robot pipetting accuracy tests. A predetermined mass (target value) of each test substance (water, ethanol, aqueous ammonia solution) was pipetted three times into a beaker. As a rule, it was determined that the target value had to be reached only by an addition process. Subsequent correction, e.g., by removing excess mass, was excluded. This procedure corresponds to that of the robotic system. For each test, the exact mass and the time required for it were measured. The adding time was defined as the time required only for the adding process itself. The preparation of auxiliary equipment (balance, beaker, pipette) or the setup of the workplace is not included in this time. Analogously, the time required for equipping and commissioning the robotic system was not included, neither. A mass of 60.0 g was set as the target value for water and ethanol, and 10.0 g for the 25 wt% aqueous ammonia solution.

### Characterization

The hydrodynamic diameter of the silica NPs was determined by using a ZetaSizer ZS (Malvern Instruments, United Kingdom). The measurements were performed in a triplicate run with at least 12 measurements per run at 25 °C. The particle size of the silica NPs synthesized by the robot was determined by using a Supra 25 SEM (Zeiss, Germany) at 2 kV (field emission). The samples were placed on a carbon pad on an SEM sample holder.

### Statistic

The results are expressed as mean ± SD. The particle size distributions of silica NPs obtained from DLS represent an average of three individual measurements of the same batch. The analysis of DLS data as well as dosing experiments was done with OriginPro 2019 (64-bit) 9.6.0.172.

## Supplementary Information

Supplementary Video 1.

Supplementary Information.

## Data Availability

We confirm, that all data generated or analyzed during this study are included in this published article and its supplementary information files.
