# Supplementary material for: Establishing and testing a robot-based platform to enable the automated production of nanoparticles in a flexible and modular way
Source: Sci Rep. 2023 Jul 15;13:11440. doi: 10.1038/s41598-023-38535-6 (PMC10349877; doi:10.1038/s41598-023-38535-6)
Supplement: Supplementary file 2 — Supplementary Information. [file 41598_2023_38535_MOESM2_ESM.pdf]

Supplementary Materials

Establishing and testing a robot-based platform to enable the automated production of nanoparticles in a flexible and modular way

S. Dembski,[1,2]* T. Schwarz,[1] M. Oppmann,[1] S. T. Bandesha,[1] J. Schmid,[3] S. Wenderoth,[1] K. Mandel,[4,1] J. Hansmann[5,1]

[1]Fraunhofer Institute for Silicate Research ISC, 97082 Würzburg, Germany
[2]University Hospital Würzburg, Department of Tissue Engineering and Regenerative Medicine TERM, 97070 Würzburg, Germany
[3]Goldfuß engineering GmbH, Laboratory Automation, 72336 Balingen, Germany
[4]Department of Chemistry and Pharmacy, Friedrich-Alexander University Erlangen-Nürnberg (FAU), 91058 Erlangen, Germany
[5]University of Applied Sciences Würzburg-Schweinfurt, Faculty of Electrical Engineering, 97421 Schweinfurt, Germany

Manufacturing of silica nanoparticles (NPs): Process documentation of single manual synthesis steps

Used chemicals

The following protocol describe the process of the production of silica NPs. Basically, NPs with a size from 20 nm to 1000 nm can be produced. In this protocol, the description to produce particles with a size of 200 nm is given. For different sizes the parameters must be adapted accordingly. The used chemicals are depicted in Fig. S1.

- Ethanol 99%
- Aqueous ammonia solution (25 wt%)
- Tetraethyl orthosilicate (TEOS, 98%)
- Deionized water

A

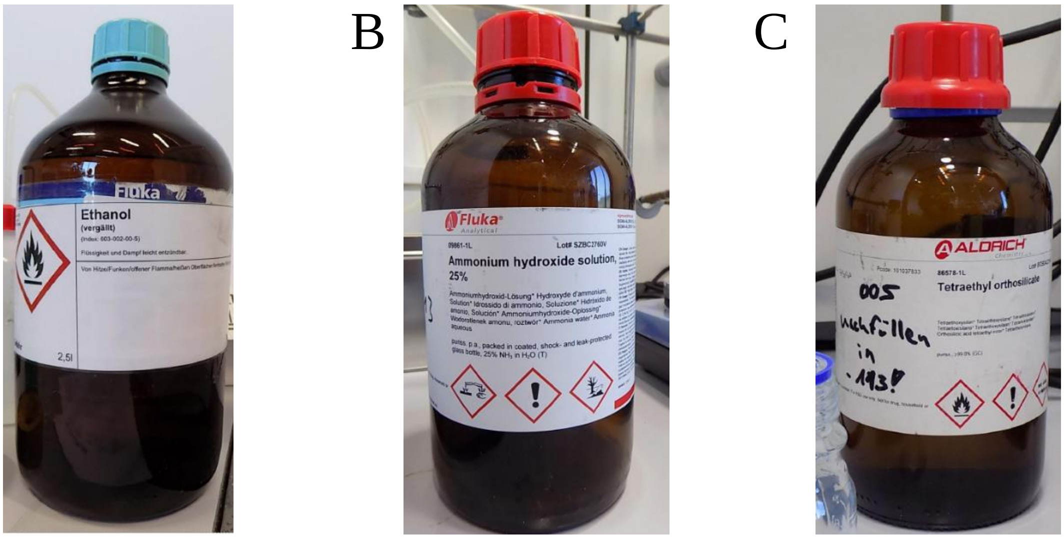

Figure S1. Used chemicals, (A) ethanol, (B) aqueous ammonia solution, and (C) tetraethyl orthosilicate (TEOS)

Step by step instruction

1. Add a magnetic stir bar of at least 2 cm in size into a glass bottle of 100 mL (Fig. S2). For larger volumes, bigger magnetic stir bars are required.

A B

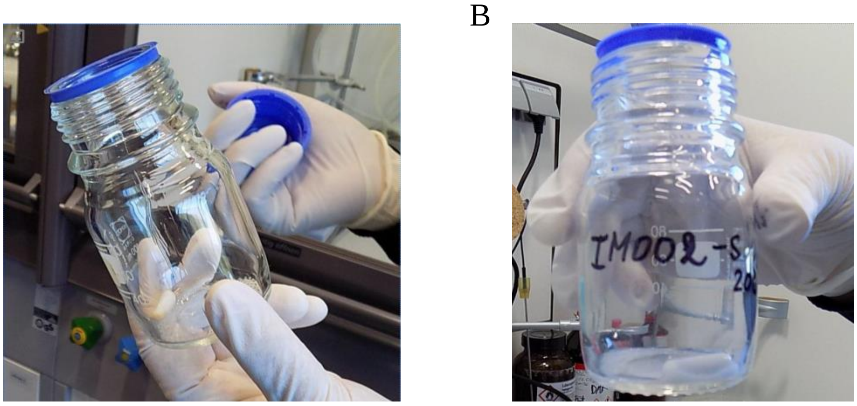

Fig. S2. Glass bottle for the preparation of the mixture, (**A**) without and (**B**) with magnetic sti

2. Add all chemicals into the glass bottle. Use a balance to check the correct amount of each chemical (Fig. S3A).
    a. Deionized water 3 g, required accuracy ± 0.01 g
    b. Ethanol 47.36 g, required accuracy ± 0.01 g
    c. Add aqueous ammonia solution 7.2 g, required accuracy ± 0.01 g

3. Then, stir the liquid with a magnetic stirrer (approx. 1 min at 350 rpm) (Fig. S3B and S3C). The speed of the stirring depends on the amount of liquid. Use a higher speed for larger vessels.

A B C

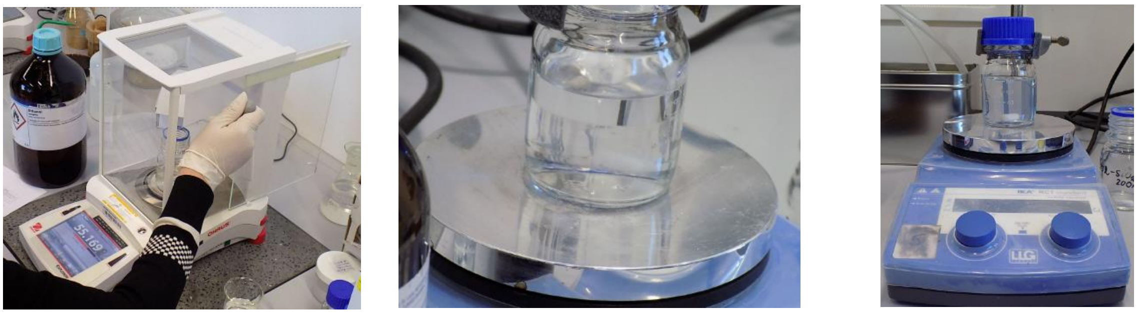

Figure S3. Adding and mixing of chemicals, (A) dosing of the chemicals by weighing, (B and C) Mixing with the magnetic stir bar.

4. Heat the mixture to 60 °C in oil bath under vigorous stirring.

5. Prepare 3 g TEOS in an extra vessel.

6. Increasing the stirring speed to 450 rpm.

7. Add TEOS to the glass bottle containing the ethanol, aqueous ammonia solution, and water.

8. Stir the mixture for 2 h at 60 °C in the oil bath. Fig. S4 shows the flask after the incubation.

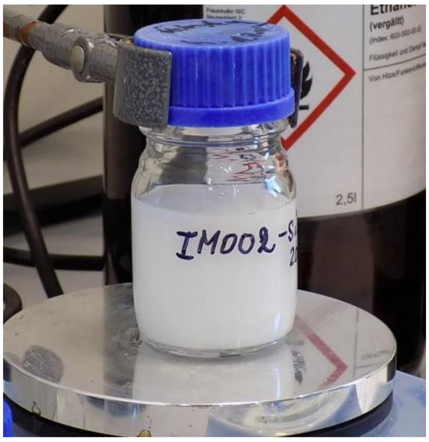

Figure S4. Mixture in the glass bottle after 2 h reaction time.

Purification of NPs

9. Divide the mixture on the four centrifuge tubes and clean the rest with ethanol (Fig. S5).

A B

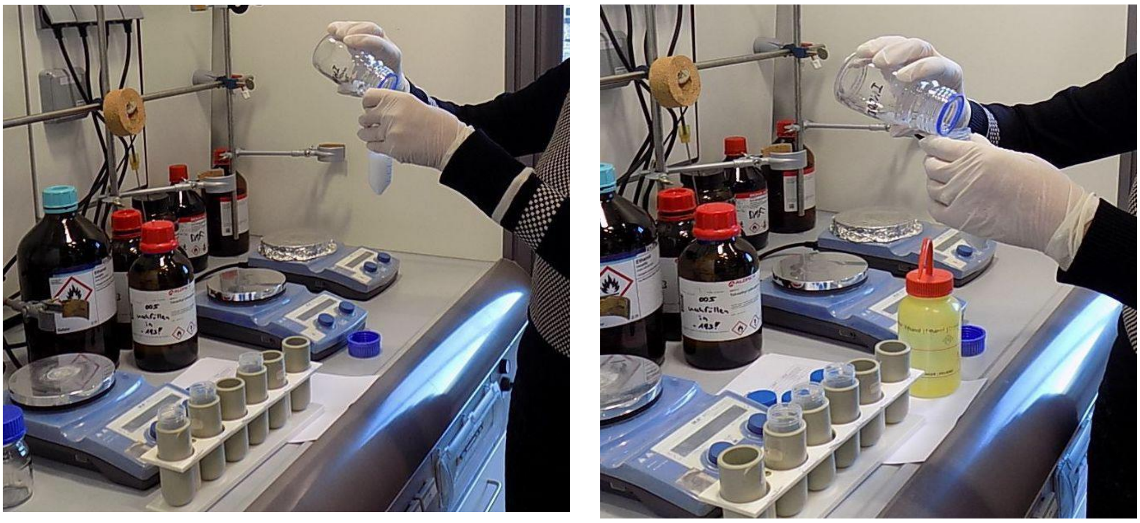

Figure S5. Dividing the mixture on the four tubes (A); cleaning of the bottle with ethanol (B).

10. Tare the tubes with ethanol on the balance by weighing (Fig. S6).

A B

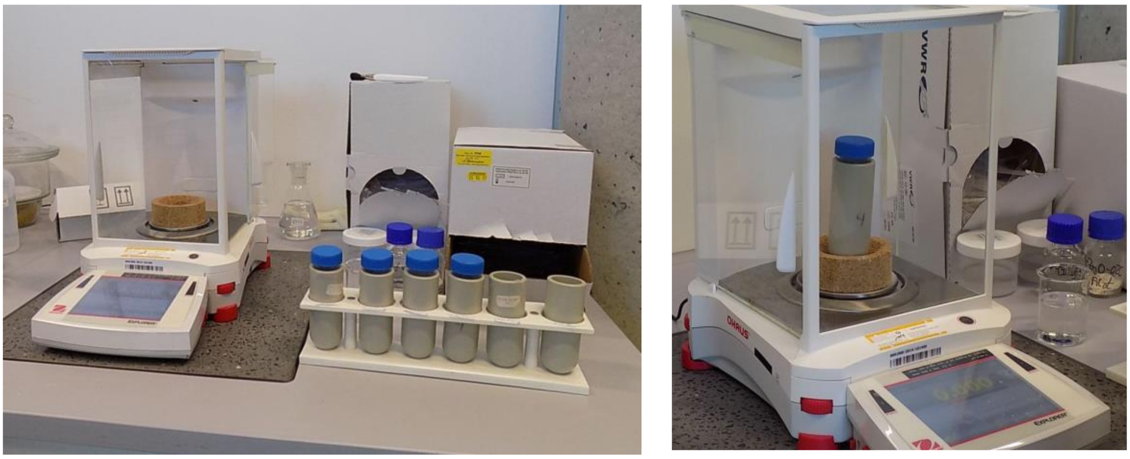

Figure S6. Taring of particle mixture with ethanol on the balance.

11. Put the tubes into centrifuge and centrifuge for 8 min at 5000 rpm (Fig. S7).

A B

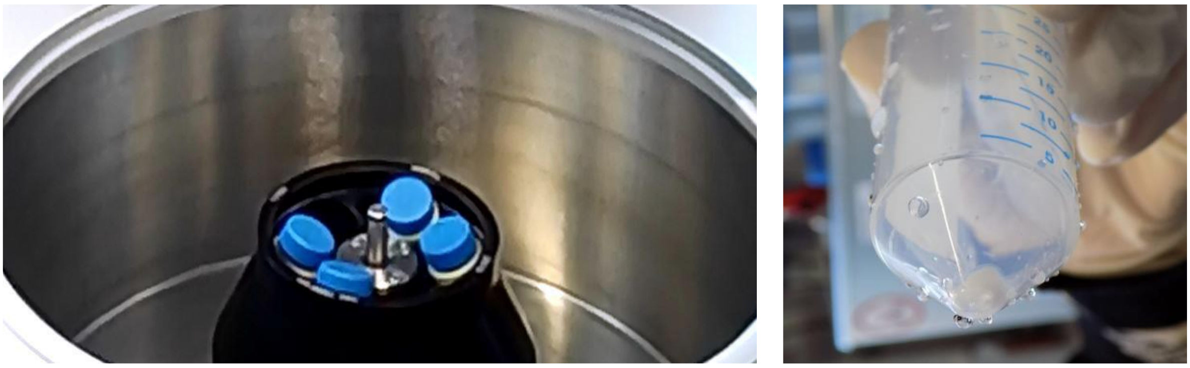

Figure S7: Tubes in the centrifuge (A). Nanoparticle pellet after centrifugation (B).

12. After centrifugation decant the supernatant, add water to all tubes and redisperse pellet by ultrasonication (Fig. S8).

A B

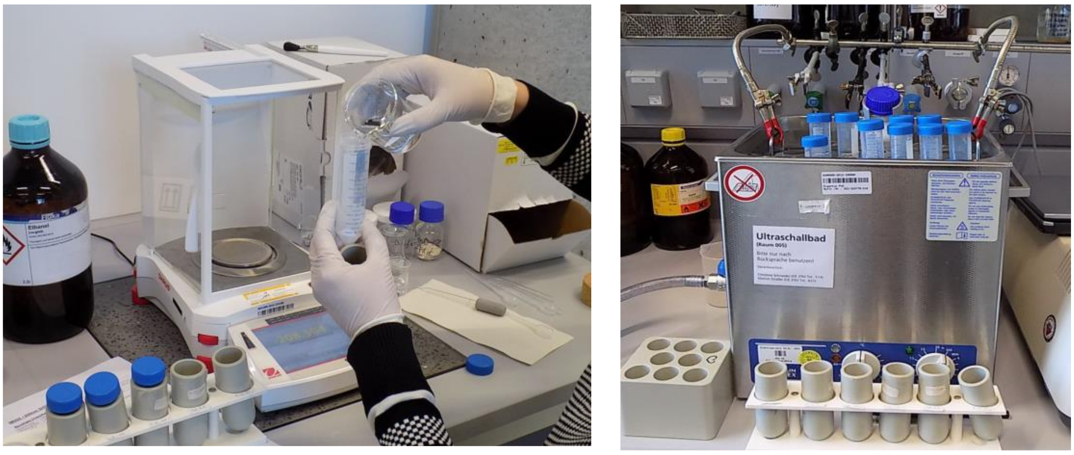

Figure S8. Decantation and redispersion of nanoparticle pellet in water (A). Dispersing by ultrasonication (B).

13. Repeat the centrifugation and redispersing steps 4 times.

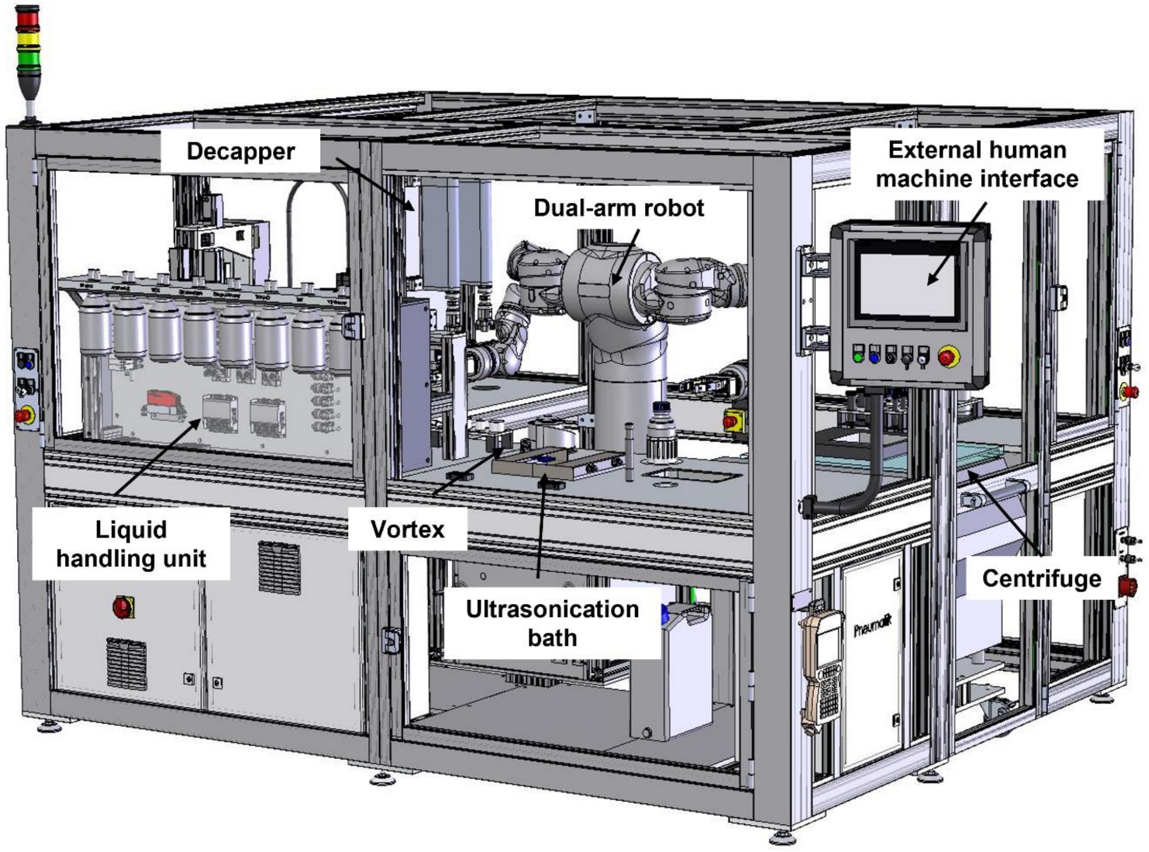

Figure S9. Computer aided design (CAD) model showing the installation of the dual-arm robot in a tailored housing made from aluminum profiles. The housing is closed and can be accessed through specific ports that facilitate maintenance and supply with materials. Inside the cell, a dual-arm robot, liquid handling unit, decapper, vortex, ultrasound device, and a centrifuge are installed. A human machine interface allows the user to control the system.

Laser scanning microscopy of silica NPs

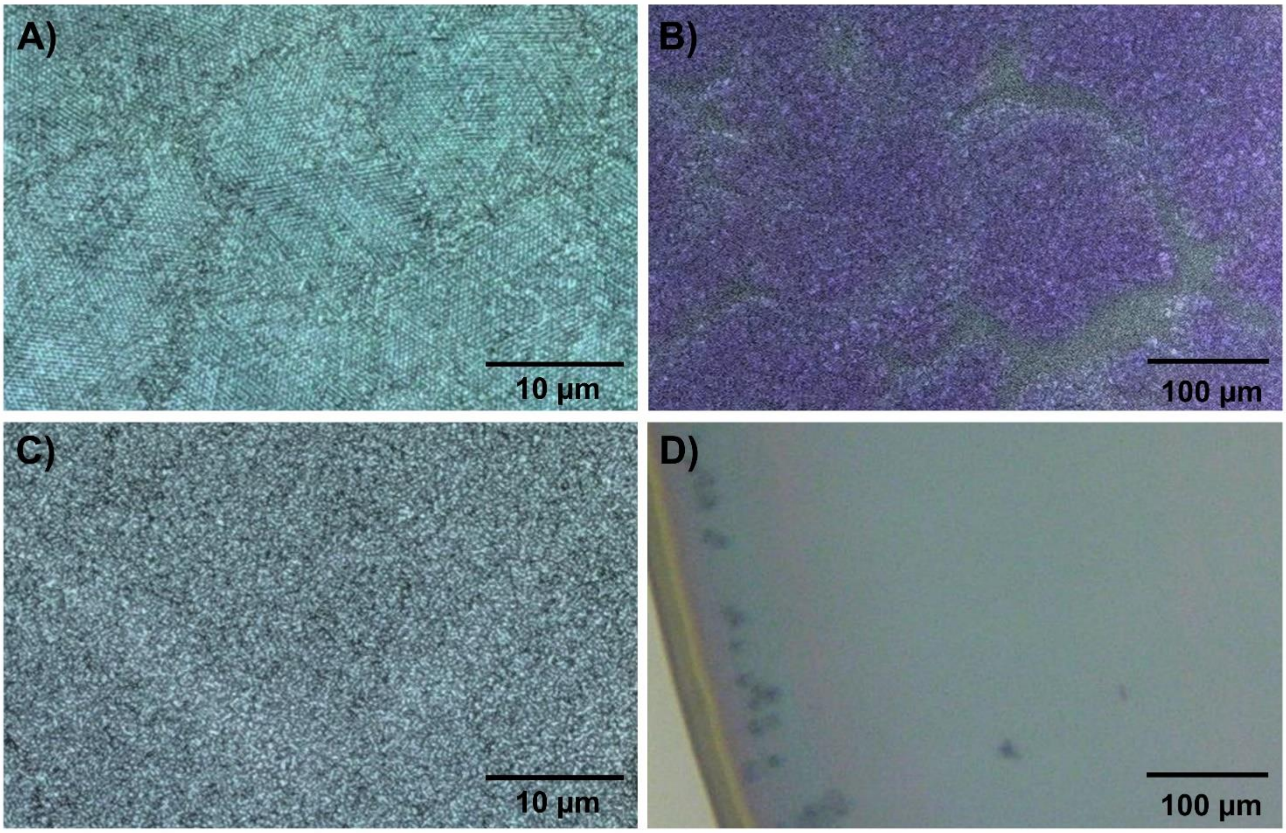

Figure S10: Example for blue photonic coloration as an occurring property of automated prepared monodispersed silica NPs. Dried automated prepared silica NP layer on the glass slide under laser scanning microscope (**A, B**); Dried manual prepared polydisperse silica NP layer on the glass slide under laser scanning microscope (**C, D**).

Movie S1: Automated nanoparticle production; demonstration of plant infrastructure including single process steps.
